# A Review on Micromixers

**DOI:** 10.3390/mi8090274

**Published:** 2017-09-09

**Authors:** Gaozhe Cai, Li Xue, Huilin Zhang, Jianhan Lin

**Affiliations:** 1 Key Laboratory of Agricultural Information Acquisition Technology (Beijing) of Ministry of Agriculture, China Agricultural University, 17 East Qinghua Road, Beijing 100083, China; gaozhe@cau.edu.cn (G.C.); li_xue@cau.edu.cn (L.X.); huilinzhang@cau.edu.cn (H.Z.); 2 Modern Precision Agriculture System Integration Research Key Laboratory of Ministry of Education, China Agricultural University, 17 East Qinghua Road, Beijing 100083, China

**Keywords:** passive micromixer, active micromixer, microfluidic mixing, microfluidic device

## Abstract

Microfluidic devices have attracted increasing attention in the fields of biomedical diagnostics, food safety control, environmental protection, and animal epidemic prevention. Micromixing has a considerable impact on the efficiency and sensitivity of microfluidic devices. This work reviews recent advances on the passive and active micromixers for the development of various microfluidic chips. Recently reported active micromixers driven by pressure fields, electrical fields, sound fields, magnetic fields, and thermal fields, etc. and passive micromixers, which owned two-dimensional obstacles, unbalanced collisions, spiral and convergence-divergence structures or three-dimensional lamination and spiral structures, were summarized and discussed. The future trends for micromixers to combine with 3D printing and paper channel were brought forth as well.

## 1. Introduction

In the past decade, various microfluidic or lab-on-a-chip [1] devices have been attempted the analysis of biological and chemical targets in the fields of biomedical diagnostics, food safety control, environmental protection, and animal epidemic prevention, etc., and have received increasing attention due to their compact size, automatic operation, faster detection, less reagent, higher sensitivity and in-field use. They can generally integrate injection, mixing, reaction, washing, separation and detection onto a centimeter-level chip [2]. Micromixers, which have a considerable impact on the efficiency and sensitivity of microfluidic devices, are one of the most important components of these devices. Unlike the macro-scale fluidic devices where the mixing of fluids often relies on convection effects, mixing in the micro-scale fluidic ones is often achieved in the microchannels with external turbulences and/or special microstructures at micro-level dimensions to obtain larger surface-to-volume ratio and increasing heat and mass transfer efficiency. Besides, the flow rates of the fluids are generally very low in the microfluidic devices and the regime of the fluids in the microchannels are basically laminar flow with the Reynolds number of <1, indicating the fluid flows in parallel layers with no disruption between the layers and the mixing of the fluids is mainly dependent on diffusion with a very low mixing efficiency. For example, in a water-based (a fluid density of 1 kg/m^3^ and a viscosity of 0.001 N·s/m^2^) microfluidic system with a channel width of 100 μm and a flow rate of 1 μL/s, the Reynolds number is 0.1 and it takes 1 s for the fluids to diffuse 1 µm and 1000 s for 1 mm. Therefore, it is crucial to develop efficient micromixers to increase the mixing efficiency for the development of microfluidic systems.

The mixing efficiency is a key parameter for a micromixer. Some methods have been proposed to evaluate the mixing efficiency. A commonly used method is based on the intensity of segregation. The standard deviation of pixel intensity or point concentration was often used as the mixing index (MI) to evaluate the mixing efficiency [3] and could be expressed by

(1) $$\mathrm{MI}=\sqrt{\frac{1}{N}\overset{N}{\underset{i=1}{\sum }}{\left(c_{i}-\bar{c}\right)}^{2}}$$

where, $c_{i}$ is the point concentration/pixel intensity, $\bar{c}$ is the mean concentration/intensity, and *N* is the number of sampling points. An improved mixing index [4] based on the comparison of the standard deviation to the mean concentration/intensity was also reported and could be expressed by

(2) $$\mathrm{MI}=1-\frac{\sqrt{\frac{1}{N}\sum_{i=1}^{N}{\left(c_{i}-\bar{c}\right)}^{2}}}{\bar{c}}$$

Besides, the mixing index [5,6] based on the comparison of the standard deviation of the point concentration or pixel intensity in the mixing section to that in the non-mixing section were proposed and could be expressed by

(3) $$\mathrm{MI}=1-\frac{\sqrt{\frac{1}{N}\sum_{i=1}^{N}{\left(c_{i}-\bar{c}\right)}^{2}}}{\sqrt{\frac{1}{N}\sum_{i=1}^{N}{\left(c_{0}-\bar{c_{0}}\right)}^{2}}}$$

where, $c_{0}$ is the point concentration or pixel intensity in the non-mixing section and $\bar{c_{0}}$ is the mean concentration or intensity in non-mixing section. Another mixing index [7] based on the comparison of the integral of the point concentration or pixel intensity in the mixing and non-mixing section was also reported and could be expressed by

(4) $$\mathrm{MI}=1-\frac{\int_{0}^{H}\left|c_{i}-c_{\infty }\right|dy}{\int_{0}^{H}\left|c_{0}-c_{\infty }\right|dy}$$

where, *H* is the width of the section and $c_{\infty }$ is the complete mixed concentration (0.5).

Micromixers are often classified as active and passive mixers [8,9,10]. Active micromixers generally require external energy sources, such as electrical, magnetic, and sound fields, etc., while passive micromixers don’t require external energy input except the energy for driving the fluids and often use complex channel geometries to enhance the diffusion or chaotic advection. The structures of active micromixers are often relatively simple and the mixers are easier to control, but the requirement of external energy sources makes them more difficult to integrate. Passive mixers are much easier to integrate into microfluidic devices, but they often require complex fabrication processes.

Although two recent excellent reviews on micromixers have been reported [11,12], one of them summarized both passive and active micromixers that had been developed before 2011, and the other published in 2016 summarized recent passive micromixers. However, in recent years, both active and passive micromixers have been reported based on new principles and structures. Thus, the advances on both active and passive micromixers over the past five years were reviewed in this study.

## 2. Active Micromixers

Active micromixers depend on different external energy sources to disturb the fluids, increase the contact area, or induce the chaotic advection, thus enhancing the mixing effect. Based on the types of external energy sources, the active micromixers can be further categorized as pressure field driven [7,13,14,15,16], electrical field driven [17,18,19,20,21,22,23,24,25,26,27,28,29,30,31,32,33], sound field driven [6,34,35,36,37,38,39,40,41,42,43], magnetic field driven [44,45,46,47,48,49,50,51,52,53,54,55,56,57], and thermal field driven [58,59,60,61,62,63], etc. (Table 1).

### 2.1. Pressure Field Driven Micromixers

**Figure 1:**
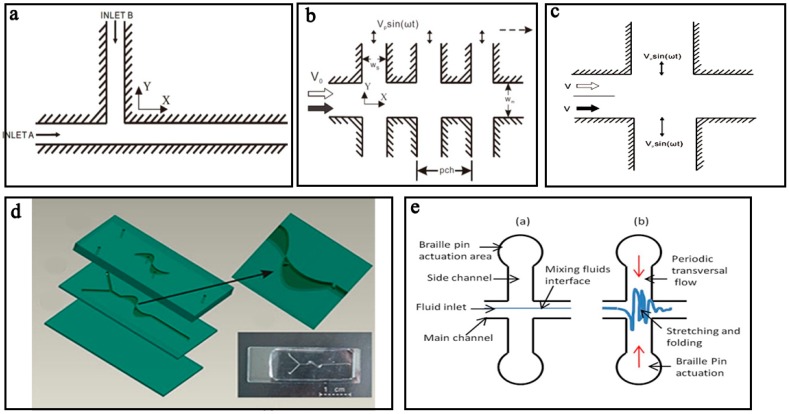
Schematic of pressure field driven micromixers with (**a**) a main channel and a side channel; (**b**) a main channel and multiple side channels; (**c**) two cross channels; (**d**) two mixing chambers, two barriers and two pneumatic chambers; and (**e**) Braille pin actuator. Reproduced with permission from [13,14,64,65].

The pressure field driven micromixers often have simple structures and consist of a main channel with a side channel (Figure 1a) [64], a main channel with multiple side channels (Figure 1b) [65], or two cross channels (Figure 1c) [66].

The typical pressure field driven micromixer is based on alternate perturbation, which was first reported by Deshmukh et al. [67] using the pulsatile flow micropumps to induce alternate perturbation on fluids in 2000. Some similar micromixers were then reported for mixing two fluids with different flow characteristics and hydrodynamic instability [15,16,68]. One common design of the pulsatile pressure driven micromixer uses two micropumps and a typical T-type channel [16]. These two pumps are used to alternately inject the fluids into the channel. The contact area of the two fluids is greatly enlarged, resulting in a better diffusion and thus a higher mixing efficiency. Additionally, Khoshmanesh et al. [69] presented a simple pressure field driven micromixer with gas bubbles. This micromixer comprises of a main channel filled with water and a side channel connected to a hydrodynamic actuator by a feeder tube to generate bubbles. By oscillating the bubbles at a given frequency, the displacement of bubbles could enhance the mixing efficiency within the main channel.

For the pulsatile pressure driven micromixers, the phase difference of the alternating voltages applied on the two pulsatile pumps has a great impact on the mixing efficiency. Sun and Sie [70] developed a pulsatile pressure driven micromixer with a diverging T-type channel. The phase differences ranging from 0 to π were compared and it was found that the mixing efficiency could reach 95% at the optimal phase difference of 0.5π and the optimal diverging angle of 55°.

Another typical pressure field driven micromixer is based on oscillatory perturbation, which is generated by extruding and vibrating the side channels [4,7,13,14,71]. Lee et al. [71] presented a typical micromixer with periodic pressure perturbations in the side channels to fold and stretch the main stream. A common pneumatic micromixer is shown in Figure 1d [13], which was comprised of an S-shaped structure with two mixing chambers, two barriers and two pneumatic chambers. With a pumping frequency of 50 Hz, the micromixer could achieve efficient mixing over a wide range of flow rates from 1 μL/min to 650 μL/min. CdS quantum dots were prepared successfully by this micromixer and showed a sharper absorption spectra than those prepared by using the conventional method. Similarly, Tekin et al. [4] used two pairs of chambers on both sides of the main channel to induce an unidirectional flow in the micromixer. By releasing the chambers on one side and at the same time pressurizing the chambers on the other side periodically, complete mixing could be achieved in 350 ms (milliseconds). As shown in Figure 1e, Abbas et al. [14] designed another interesting pressure driven micromixer in a polydimethylsiloxane chip using Braille pin actuation at a resonance frequency (10 Hz) on the side channels to stretch and fold the fluid in the main channel, thus achieving chaotic mixing. This micromixer was successfully used for a continuous dilution of a yeast cell sample by a ratio down to 1:10.

### 2.2. Electrical Field Driven Micromixers

The electrical field driven micromixers are mainly based on electro-hydrodynamic (EHD) instability [22], which often uses the motion of electrically charged fluids under an alternating current (AC) or direct current (DC) electric field to disturb the interface of the fluids.

One typical electrical field driven micromixer was presented by Huang [17]. It applied a time-periodic electric field on an electrode array to generate electro-thermal vortices at the corners of each pair of electrodes. The vortices could induce the convective diffusion and thus mix the fluids efficiently. Huang [17] used an AC signal with a peak-to-peak voltage of 6 V, a frequency of 1 MHz, and a phase shift of 180° to actuate an electrode array with an electrode width of 100 µm, an electrode spacing of 30 µm, and an electrode set of 3 in a channel with a width of 400 µm and a height of 30 µm. The mixing efficiency could achieve 94% in ~30 min. Additionally, it was found that the number of the vortices at the corners of the electrodes was reduced from 4 to 2, while the electrode width was the same as the electrode spacing. Zhou et al. [26] employed DC voltage to generate in-plane vortices in an improved microchannel with asymmetric lateral structure and earned a better mixing efficiency.

**Figure 2:**
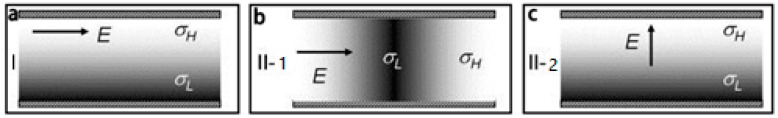
Typical base states for electro-kinetics with type I (**a**); type II-1 (**b**); and type II-2 (**c**). E and the arrow indicate the electric field and its direction, respectively; $\sigma_{H}$ and $\sigma_{L}$ indicate high- and low- conductivity regions, respectively. Reproduced with permission from [72].

Electro-kinetics (EKI) is a branch of EHD that describes the coupling of ion transport, fluid flow and electric fields and can be distinguished from EHD by the relevance of interfacial charge at solid-liquid interfaces [33,72]. Electro-kinetic flow instabilities occur under high electric fields in the presence of electrical conductivity gradient [29]. EKI has two basic types of configurations (Figure 2) [72]. For type I, the electric field is orthogonal to the conductivity gradient. For type II, the electric field is parallel with the conductivity gradient, and the net charge density has a non-trivial distribution even in the base state.

**Figure 3:**
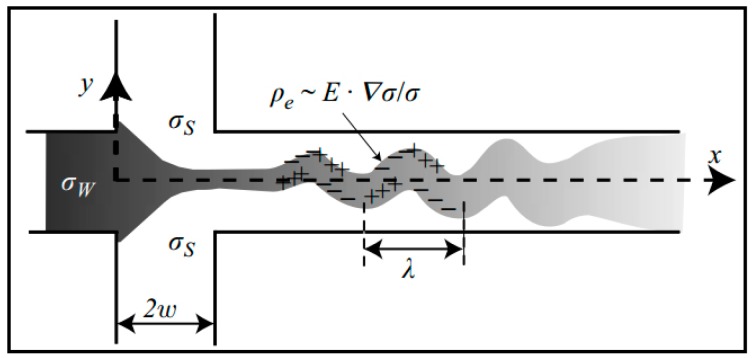
Schematic of the unstable flow in a cross-shaped microchannel with the characteristic D-shape and cross-sections of isotropic etching. *σ_s_* is the ionic conductivity of the sheath streams from top and bottom inlets. *σ_w_* is the ionic conductivity of the sample stream from the left inlet. *Pe*, *E*, *λ*, and *w* are charge density, electric field, nominal wavelength and half-width of channel, respectively. Reproduced with permission from [19].

Kumar et al. [23] demonstrated for the first time the electrokinetic instabilities of ferrofluid/water that flowed in a T-shaped channel, and found similar dynamic behaviors in the ferrofluid/water interface at various electric fields. Posner et al. [19] also presented a study on convective electrokinetic instability in a three-inlet, one-outlet electrokinetic focusing flow configuration where the center sample stream and sheath flows had different ionic conductivities (Figure 3). Electrokinetic flowed with conductivity gradients turned unstable when the electroviscous stretching and folding of conductivity interfaces grew faster than the dissipative effect of molecular diffusion. The results showed that the flow became unstable at a critical electric Rayleigh number (Ra_e,e_ = 205) for a wide range of conductivity ratios *γ* (three orders of magnitude) and applied field ratios *β*.

**Figure 4:**
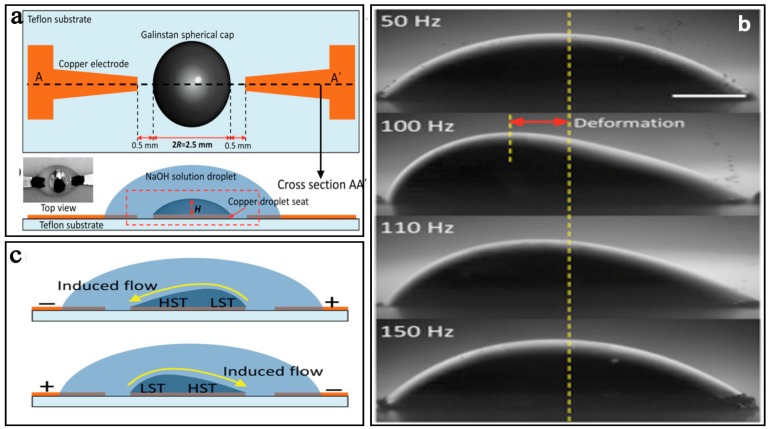
(**a**) Schematic of the micromixer with Galinstan cap; (**b**)The deformation of the Galinstan cap by applying sinusoidal signals with different frequencies and magnitudes; (**c**) Flow velocity vectors (m/s) along the Galinstan surface. Reproduced with permission from [73].

Another typical electrical field driven micromixer with liquid metal was described by Tang et al. [73]. Due to their former concept of surface tension driven flow [74], this micromixer used the same Galinstan cap (semi-spherical) placed on a circular copper substrate seat as the core of a liquid metal actuator to induce chaotic advection (Figure 4a). Under a sinusoidal AC electric field (4 V, 50 Hz to 150 Hz), periodic deformation of the Galinstan cap could be observed due to the tangential force, which pulls the surrounding liquid along the surface from the regions of low surface tension (LST) to the regions of high surface tension (HST) (Figure 4b,c). The time averaged mixing efficiency could reach 95% at the flow rate of 25 µL/min when the 4 V & 50 Hz signal was applied.

### 2.3. Sound Field Driven Micromixers

Sound field driven micromixers are based on acoustic resonant disturbance, which was first reported by Moroney et al. [75] using a Lamb-wave membrane device to enhance the mixing.

**Figure 5:**
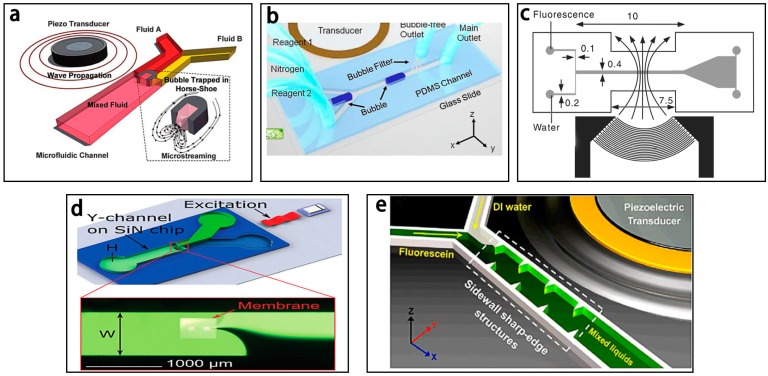
Schematic of the sound field driven with (**a**) “horse-shoe’’ structure; (**b**) nitrogen gas; (**c**) interdigitated electrodes; (**d**) vibrating membrane; and (**e**) sidewall sharp-edges. Reproduced with permission from [6,36,43,79,82].

One typical sound field driven micromixer is based on the use of microbubbles to achieve fast convective mixing [76]. The bubble-based acoustic micromixer with a microstreaming flow field was first reported by Liu et al. [77,78] and it was demonstrated to successfully accelerate the rate of the DNA hybridization process (~5 times faster). However, it generated too many bubbles in the channel. To overcome this drawback, Ahmed et al. [79] developed a single bubble based acoustic micromixer shown in Figure 5a and verified that it could realize complete mixing in 7 ms by trapping the air bubbles in the “horse-shoe’’ structure to induce microstreaming in the microchannel.

Some other bubble-based sound micromixers have also been reported. Ozcelik et al. [38] utilized the surface roughness of the polydimethylsiloxane (PDMS) microchannel’s sidewalls to cavitate the bubbles and obtained excellent mixing efficiency (92%) for high-viscosity fluids at a low Reynolds number of 0.01 in less than 100 ms. Wang et al. [80] presented another bubble based micromixer, which was made up of a 300 μm thick dry adhesive layer sandwiched between two 2-mm-thick polymethylmethacrylate layers. A nozzle-shaped chamber with an acoustic resonator profile was developed in the adhesive layer for generating the bubbles in the microchannel when the piezo-electric disk under the chamber was actuated at the frequency range of 1–5 kHz. Besides, nitrogen gas was reported for generating bubbles to develop bubble based micromixers. As shown in Figure 5b, the nitrogen gas was injected in the center of two reagents in the microchannel and microstreaming was generated for mixing these two reagents [43]. The proposed micromixer could mix two highly viscous fluids (95.9 mPa·s) in the presence of an acoustic field within 50 ms with an excellent mixing efficiency of ~93% at a low *Re* number (~0.01).

Another typical sound field driven micromixer is based on a surface acoustic wave (SAW), which is an acoustic wave traveling along the surface of a solid material [81]. As shown in Figure 5c, Luong et al. [82] reported the use of focusing interdigitated electrodes instead of traditional parallel interdigitated electrodes to concentrate the acoustic energy. The SAW was generated by the interdigitated electrodes deposited on the piezoelectric substrate to induce mixing due to the disturbance of the transversal acoustic streaming. The mixing efficiency of 90% was obtained with the peak-to-peak voltage of 80 V and the Peclet number of 74.4 × 10^3^. Besides, vibrating membrane, micro-pillars and sidewall sharp-edge were also reported in the development of the continuous-flow micromixers. As shown in Figure 5d, Phan et al. [6] developed a vibrating membrane with a hole to generate strong streaming vortices in the channel. Besides, micro-pillars was also utilized to realize homogeneous mixing in 6 s by Oever et al. [40] in a centimeter-scale acoustic micromixer. Huang et al. [36] reported the oscillation of sidewall sharp-edges to induce an acoustic streaming to achieve excellent mixing in 180 ms (Figure 5e).

### 2.4. Magnetic Field Driven Micromixers

Magnetic field driven micromixers are mainly based on magneto-hydrodynamics (MHD) and magnetic stirring.

**Figure 6:**
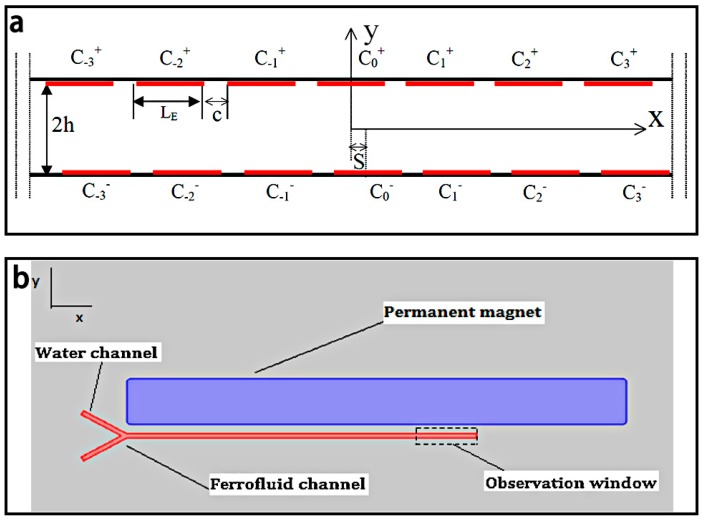
(**a**) Schematic of the magneto-hydrodynamic micromixer. *L_E_* is the width of the electrodes, *c* is the width of the space between two adjacent electrodes, *S* is the offset of two facing elecrodes, and 2*h* is the width of the conduit; (**b**) Schematic of the ferrofluid-based magnetic micromixer with Y-shaped channel. Reproduced with permission from [57,83].

Magneto-Hydrodynamic micromixers often utilize AC or DC electric fields and magnetic fields to apply Lorentz forces on the magneto-fluids, which can induce secondary flows for stirring and mixing. One typical magneto-hydrodynamic micromixer [83] is shown in Figure 6a, which consisted of a conduit filled with an electrolyte solution, and individually controlled electrodes patterned along its double sidewalls. When the micromixer was placed in a uniform magnetic field, it could serve as both a mixer and a pump. Recently, ferrofluid was extensively employed for the studies on magnetic micromixers [48,49,53,56,57]. A ferrofluid-based microfluidic magnetic micromixer developed by Cao et al. [48] using a hybrid magnetic field generated by some micro-magnets and an external AC uniform magnetic field to apply periodic magnetic forces on the ferro-fluid, thus achieving a high mixing efficiency (97%) in 8 s at a distance of 600 μm from the mixing channel inlet. As shown in the Figure 6b is another ferrofluid-based magnetic mixer developed by Nouri et al. [57] using a Y-shaped microchannel with a permanent magnet to mix deionized water and Fe_3_O_4_ ferrofluid. The ferrofluid migrated from one bottom side of the channel to the top side under the magnetic field generated by the permanent magnet, resulting in the mixing of the two fluids.

**Figure 7:**
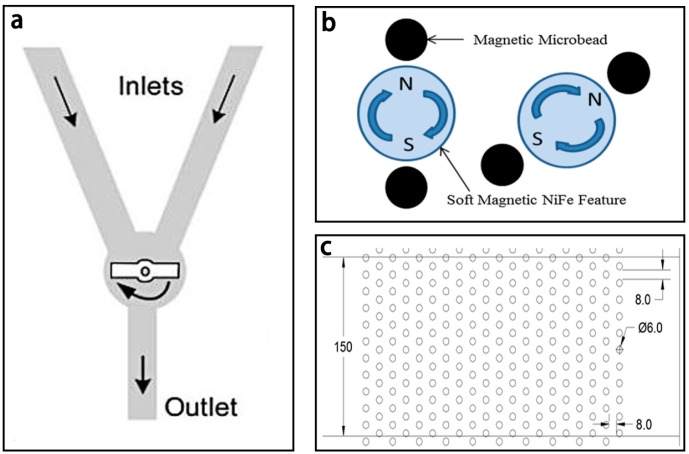
(**a**) Schematic of the magnetic stirring micromixer; (**b**) Schematic of the magnetic microbeads attracted to the poles of the NiFe feature; (**c**) Schematic of the microfluidic channel with its floor patterned with NiFe features. Reproduced with permission from [54,84].

Magnetic stirring micromixers often used magnetic stirrers driven by external rotating magnetic fields to mix the fluids in the chamber. A typical magnetic micromixer is shown in Figure 7a [84]. The millimeter-sized magnetic stirrer was controlled by the rotating field to stir the fluids in the channel and the fluids could reach total mixing within seconds. Veldurthi et al. [50] studied the simulation of this stirring micromixer at different rotating speeds of the magnetic stirrer over a wide range of flow rates, and the results showed that the maximum mixing efficiency (~90%) was achieved at 1500 rpm. Additionally, the rifampicin drug was successfully loaded on TiO_2_ nanoparticles by this micromixer. Microbeads were also used as stirrers in microchannels [45,51,54]. Owen et al. [54] proposed a micromixer with short channel lengths (270 μm) by an array of rotating magnetic microbeads (Dynabeads M-280, 2.8 μm diameter), which was attracted to the poles of the NiFe feature driven by an external magnetic field (Figure 7b,c). Complete mixing could achieve in 2.5 to 9 s depending on different flow velocity and specific biological particles in the fluid could be captured by the magnetic microbeads with different functional groups. 

In addition, artificial cilia with embedded magnetic particles driven by a homogeneous magnetic field was used in a simple T-shape channel to realize the mixing of two highly viscous fluids [85]. In this micromixer, a high mixing efficiency of 86% was achieved when the figure-of-eight trajectory of artificial cilia was generated by three rolls of magnetic coils.

### 2.5. Thermal Field Driven Micromixers

**Figure 8:**
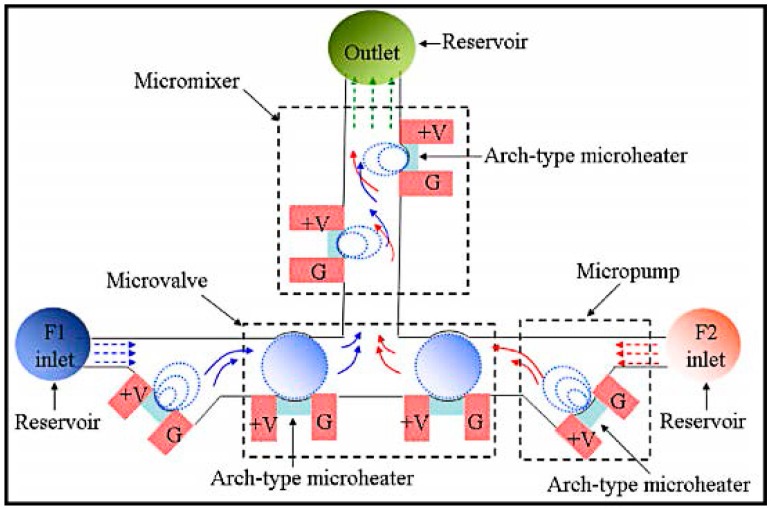
Schematic of the microfluidic system including microchannel, micro-valve, micro-pump and micromixer. Reproduced with permission from [61].

Thermal field driven micromixers are often dependent on the use of thermal bubbles for mixing. Huang [61,86] presented a thermal bubble actuated microfluidic chip with microvalve, micropump and micromixer, based on a simple process with silicon-onisolation (SOI) wafer (Figure 8). The size of thermal bubbles can be controlled at flow rate of less than 4.5 μL/s. When an AC signal at high frequency was applied to the micro-heater, the thermal bubbles could grow periodically and collapse rapidly, thus generating turbulent flow in the fluids and increased mixing efficiency.

**Figure 9:**
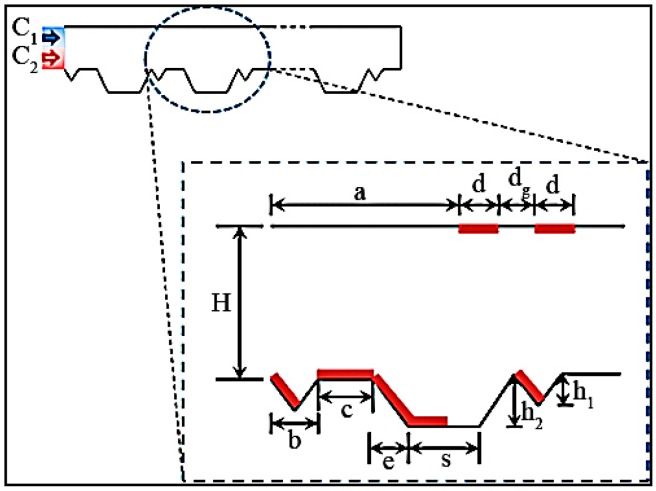
Schematic of the alternating current electrothermal micromixer. Reproduced with permission from [63].

Micromixer driven by electrothermal effect also involves lots of multi-physics phenomena which could be used in micromixers [59,60,62]. Recently, Kunti et al. [63] proposed an alternating current electrothermal micromixer consisted of eight pairs of asymmetric electrodes with AC voltage. As shown in Figure 9, in this micromixer waviness of the floor increased the contact area between two fluids and lateral vortex pairs were generated by symmetric electrode pairs located on the top wall. A mixing efficiency of 97.25% can be achieved under a flow rate of 1.794 μm^2^/min.

### 2.6. Other Field Driven Micromixers

**Figure 10:**
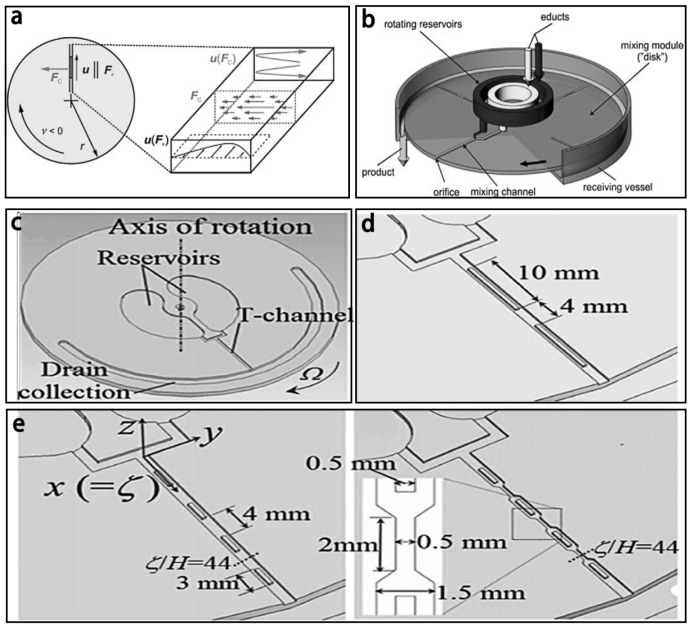
(**a**) The force analysis: *F_c_* is the Coriolis force, *F_v_* is the centrifugal force, *u*(*F_c_*) is the transverse of fluids; (**b**) Schematic of the centrifugal mixer; (**c**) Schematic of the microchannels; (**d**) with two obstructions; and (**e**) with four obstructions. Reproduced with permission from [90,91].

Centrifugal forces can also enhance mixing and has been used in lots of micromixers [87,88,89]. Haeberle et al. [90] reported a centrifugal micromixer, relying on the Coriolis force induced by the rotated plate to drive and mix the fluids (Figure 10a). Base on the centrifugal micromixer, Leung et al. [91] investigated the mixing efficiency of different rotating radial microchannels with various obstructions and/or width-constriction geometries (Figure 10b–e). The experimental results showed that transverse flow in the microchannel was highly increased due to the obstruction with constriction (OWC) configuration, local centrifugal acceleration, and Coriolis acceleration. Moreover, for the rotating OWC (obstruction follow by width-constriction) channel, the mixing efficiency could reach 95% at the distance of 30 mm from inlet when the rotating rate was 73 rad/s, which was much more than those of the stationary OWC channel, the rotating unobstructed/obstructed channel, and the rotating width-constricted channel.

## 3. Passive Micromixer

Passive micromixers—also called static micromixers—are based on the structure of the microchannels to enhance molecular diffusion and chaotic advection for efficient mixing [92]. There is an excellent 2004 review focused on passive micromixers by Nguyen et al. [8], yet there are many new passive micromixers developed recently by scientists. According to the dimensions of the structure, passive micromixers can be sub-classified as either three-dimensional (3D) and two-dimensional (2D). Over the past five years—as shown in Table 2—many new passive micromixers based on the structure of T-type [93], Zigzag [94], and Serpentine [3], etc., have been reported. 

### 3.1. 2D Passive Micromixers

2D passive micromixers with simple planar structures such as obstacles, unbalanced collisions, convergence–divergence channels, and spiral channels etc. are easy to fabricate with lithography method and generate chaotic advection due to the special shape of the channel.

#### 3.1.1. Obstacle Based Micromixers

The obstacle based micromixers are mostly combined with various embedded grooves or barriers with different shapes and heights. One typical obstacle based micromixer with straight grooves was first proposed by Stroock et al. [119]. The experimental results showed that these straight grooves aroused a secondary flow in the channel and a good mixing over a wide range of Reynolds numbers (0 < *Re* < 100). Howell et al. [120] improved this design by placing grooves in both the top and bottom of the channel. Besides, Hossain et al. [121] further optimized this micromixer, and simulation results showed that the best mixing efficiency could reach 91.7%.

**Figure 11:**
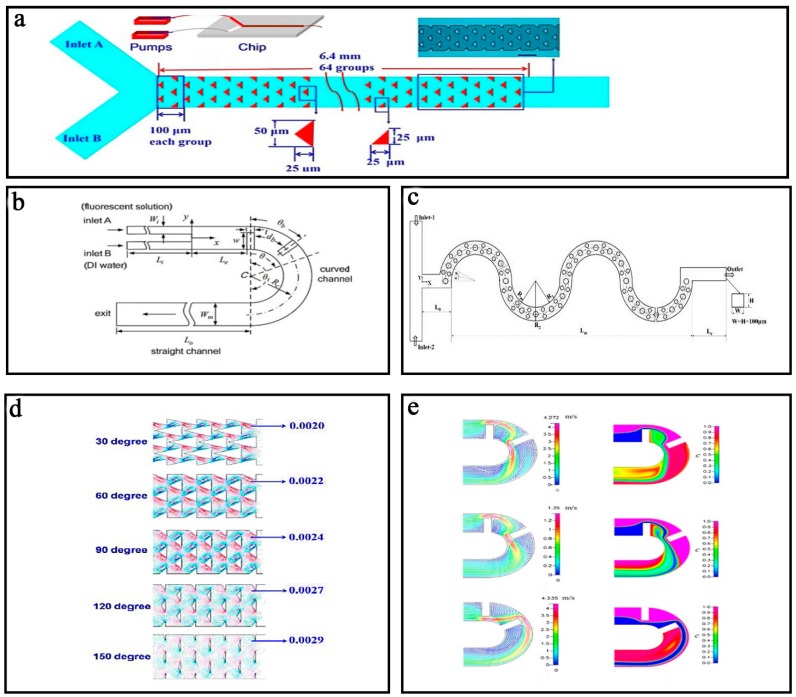
Schematic of the obstacle based micromixer (**a**) with triangle barriers, (**b**) with radial barriers, and (**c**) with cylindrical barriers; (**d**) The simulation results of the obstacle based micromixer with triangle barriers; (**e**) The simulation results of the obstacle based micromixer with radial barriers. Reproduced with permission from [97,98,128].

Another typical obstacle for these micromixers is based on the barriers in the channels. Bhagat et al. [122] studied the effect of the barriers’ height and shape on the mixing efficiency. The simulation results showed that the mixing efficiency increased when the higher barriers were used. Specifically, when the barriers had the same height as the channel the mixer was called as a split-and-recombine (SAR) one and had the best mixing efficiency. For the stepped-diamond-shaped barriers, the efficiency could reach 77%. Some similar researches [123,124] also proposed the numerical and experimental investigation on comparing the mixing behaviors of microchannel that was shaped with various kinds of barriers. They showed that by increasing the number and length of rectangular barriers can potentially enhance the mixing effect within a short mixing length in microchannels. As shown in Figure 11a, Wang et al. [97] fabricated a passive micromixer containing 64 groups of triangle barriers with excellent mixing efficiency for Reynolds numbers in the range of 0.1 to 500. Both simulation and experimental results showed that the bigger apical angles and the more groups of the triangle barriers led to the better mixing efficiency and the best mixing efficiency could reach 91.2% (Figure 11d).

Many studies [125,126,127] have demonstrated that the curved channel based micromixer, without obstacles, could not achieve a high mixing efficiency unless it had a high Reynolds number. To improve the mixing efficiency of the curved channel based micromixer at the low Reynolds number, Tsai et al. [128] proposed a planar micromixer based on multidirectional vortices in the curved channel with two radial barriers of 40 μm thick and 97.5 μm long (Figure 11b). The effects of the position and size of the radial barriers were studied (Figure 11e), and it was found that the presence of the Dean vortices [129] generated by the curved channel and the expansion vortices produced by the barriers led to a fine mixing efficiency of ~72% at *Re* = 81 in a very short length (~4.25 mm). Different from the above-mentioned curved channel based micromixers, Afroz Alam et al. [98] presented a new one employing several cylindrical barriers in the curved microchannel (Figure 11c). The barriers in the curved microchannel could generate secondary flows and SAR flows, resulting in a high mixing efficiency of 88% at both low and high Reynolds numbers (*Re* = 0.1 and 15 ≤ *Re* ≤ 60). 

#### 3.1.2. Unbalanced Collision Based Micromixers

**Figure 12:**
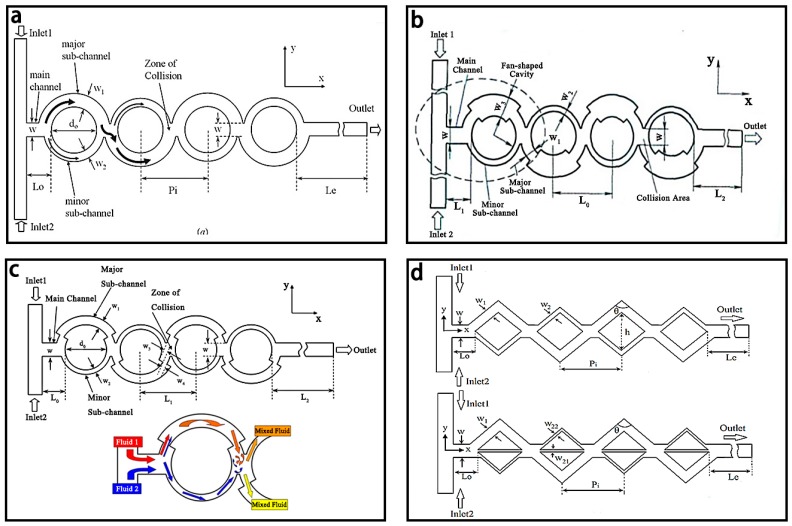
Schematic of the (**a**) unbalanced collision based micromixer; (**b**) unbalanced collision based micromixer with fan-shaped cavity in major sub-channel; (**c**) unbalanced collision based micromixer with stagger structure; (**d**) unbalanced collision based micromixers with two-split rhombus channel and with three-split rhombus channel. Reproduced with permission from [95,96,130,131].

The unbalanced collision based micromixers are often dependent on the asymmetric structure of the channel or the different flow rate of the fluids. One typical unbalanced collision based micromixer was shown in Figure 12a and based on the concept of unbalanced splits and cross-collisions of the fluids, which was first presented by Ansari et al. [130]. The mixing was mainly due to the combined effect of unbalanced collisions and Dean vortices. When *w*1/*w*2 = 2.0, the best mixing efficiency of 65% for Reynolds numbers ranging from 10 to 80 could be obtained. As shown in Figure 12b, Xia et al. [131] developed an unbalanced circular micromixer using fan-shaped cavities in the major sub-channel to generate convergent-divergent structures with the mixing efficiency of 78%. The stagger structure was used at the corner of the major sub-channel in the previously mentioned mixer with a higher mixing efficiency of 86% (Figure 12c) [96].

Moreover, a similar micromixer with three unbalanced rhombic sub-channels was developed by Hossain [95] and the simulation results showed that the mixing efficiency of the unbalanced three-split rhombus based micromixer (86%) had ~1.44 times than that of the two-split rhombus based one at the Reynolds number of 60 (Figure 12d).

#### 3.1.3. Spiral Based Micromixers

**Figure 13:**
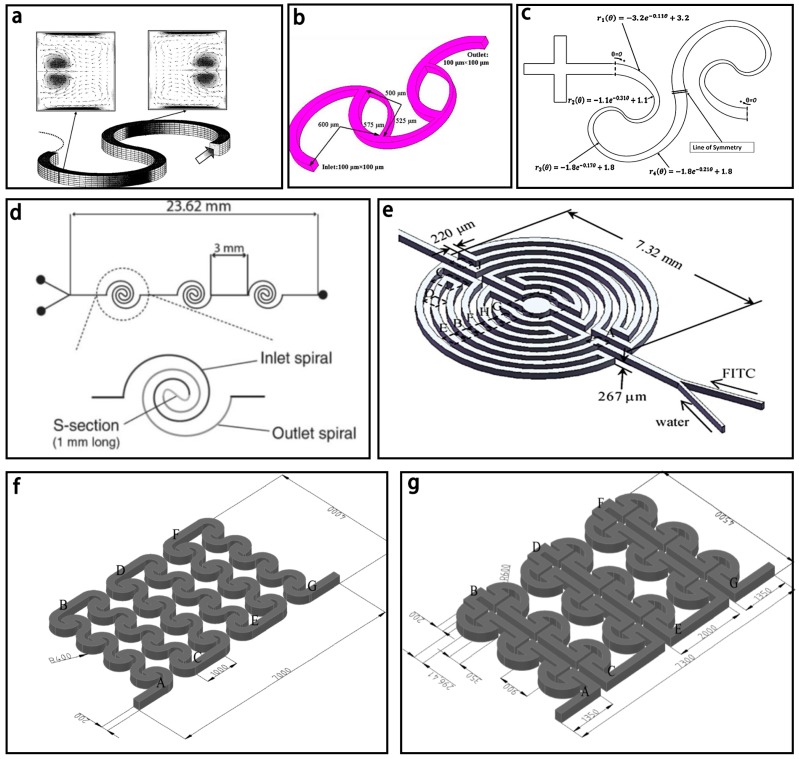
(**a**) Schematic of the spiral micromixer (The arrow indicates the flow direction); (**b**) Schematic of the parallel laminar micromixer; (**c**) Schematic of the spiral based micromixer with sequential logarithmic structure; (**d**) Schematic of the double spiral based micromixer; (**e**) Schematic of the labyrinth-like multiple spiral based micromixer; (**f**) Schematic of the spiral based micromixers with interlocking semicircles; (**g**) Schematic of the spiral based micromixers with omega-shaped channels. Reproduced with permission from [114,132,133,134,135].

The spiral based micromixer was first put forward by Schönfeld et al. [132] and is shown in Figure 13a. Subsequently, Sheu et al. [114] combined this typical spiral based micromixer with unbalanced collisions to develop a parallel laminar micromixer with two-dimensional staggered curved channels (Figure 13b). Dean vortices were formed in curved channels by centrifugal forces, and the split structures of the tapered channels resulted in the unbalanced split of the main stream and the reduction of the diffusion distance of two fluids.

Another typical spiral based micromixer is shown in Figure 13c with a maximum mixing efficiency of 86% at *Re* = 67 [99], which is significantly higher than that of the Archimedes and Meandering-S spiral based ones. Similarly, He et al. [100] reported a two logarithmic spiral based micromixer with the spiral polar angle from 0° to 180°. Because the two logarithmic spirals with the variable curvatures were parallel, secondary flows were generated to enhance mixing with the mixing efficiency of 80% at the Reynolds number of 0.2. 

An interesting double spiral based micromixer was first proposed by Sudarsan and Ugaz in 2004 [133] and is shown in Figure 13d. The mixing efficiency could reach >90% at the end of the second section. Furthermore, another interesting labyrinth-like multiple spiral based micromixer was shown in Figure 13e and a fast complete mixing within 9.8 s to 32 ms could be achieved for Reynolds numbers between 2.5 and 30 [134]. Similarly, Al-Halhouli et al. [135] presented two spiral based micromixers with interlocking semicircles and omega-shaped channels respectively (Figure 13f,g). Nearly complete mixing could be achieved for a wide range of *Re* between 0.01 and 50 in each micromixer.

#### 3.1.4. Convergence–Divergence Based Micromixer

A convergence-divergence structure of micromixers can cause expansion vortices, subsequently causing a great disturbance in the microchannel laminar flow as well as increasing the contact area between the different fluids, thereby enhancing the mixing efficiency.

**Figure 14:**
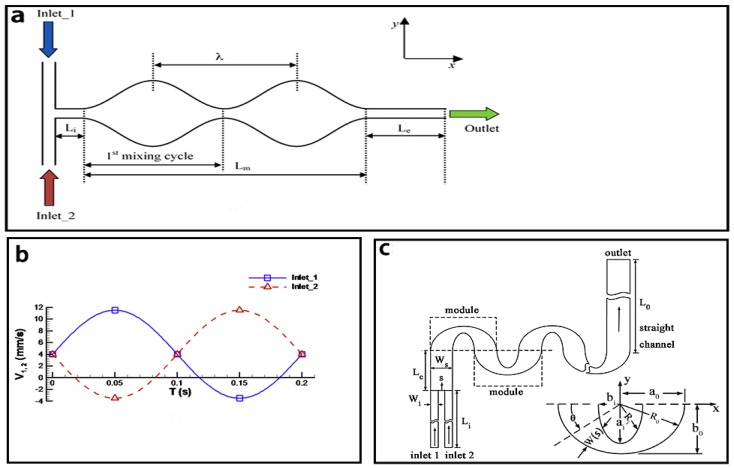
(**a**) Schematic of the convergence-divergence based micromixer; (**b**) The inlet flow velocities of the convergence-divergence based micromixer; (**c**) Schematic of the convergence-divergence based micromixer with meandering channel. Reproduced with permission from [102,136].

As shown in Figure 14a, one typical convergence-divergence based micromixer with sinusoidal walls was represented by Afzal and Kim [136]. Coupled with pulsatile flow (Figure 14b), this micromixer could achieve a mixing efficiency of 92% within two periods of the sinusoidal walls. A multi-objective optimization [101] of the Sigma micromixer [137] was proposed. Lengths of the major axis (*a*), minor axis (*b*) and the constirction width (*h*) were optimized at the *Re* = 0.91. The results showed the mixing efficiency increased with higher a/H values and lower values of b/g and h/H and a best mixing efficiency (79.1%) was reached with *a/H* = 0.75, *b/g* = 0.503 and *h/H* = 0.216. Another convergence-divergence based micromixer with meandering channel, presented by Wu and Tsai [102], showed a better mixing efficiency (80% at *Re* = 35.5) than the Sigma micromixer. Different expansion ratios, defined as *E* = *W*_max_(s)/*W*_min_(s), were studied and it showed that such micromixer with a larger expansion ratio earned a better mixing (Figure 14c). 

**Figure 15:**
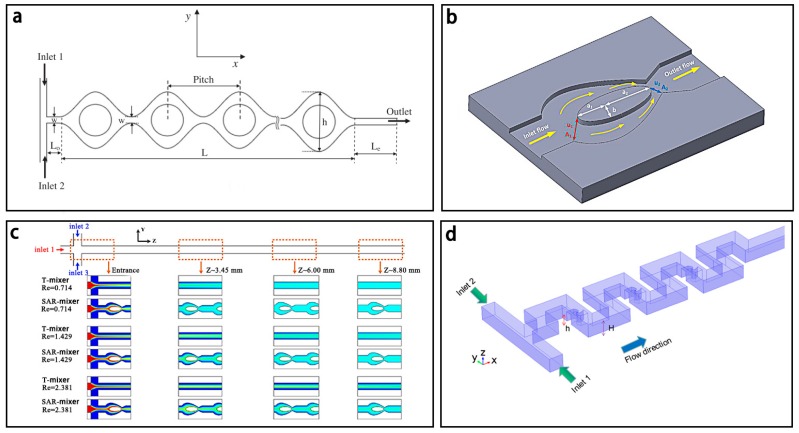
(**a**) Schematic of the convergence-divergence based micromixer with split-and-recombine structure; (**b**) Schematic of the convergence-divergence based micromixers with the ellipse-like micro-pillars; (**c**) Comparison of the mixing between the ellipse-like micro-pillars mixer and the T-channel mixer; (**d**) Schematic of the convergence-divergence based micromixers with two-dimensional serpentine channel and based on topology optimization method. Reproduced with permission from [103,104,105].

Afzal et al. [103] combined the split-and-recombine structure with the convergence-divergence walls to generate secondary flows (Figure 15a). The results showed a decent mixing efficiency of 95% could be achieved with Reynolds numbers ranging from 10 to 70. Tran-Minh et al. [104] proposed a combination of the planar split-and-recombine structure with the ellipse-like micro-pillars and it was successfully used for continuous mixing of human blood (Figure 15b). The optimal parameters (a_1_:a_2_:b = 5:6:4) for the ellipse were investigated with a high mixing efficiency of >80%, which was better than that of the T-channel mixer (Figure 15c). Recently, several convergence–divergence based micromixers, which were transformed from the typical two-dimensional serpentine channel and based on topology optimization method, were reported by Chen and Li [105]. In these micromixers, convergence-divergence structures were set at the center of the channel with the obstacles at different height (Figure 15d), The results showed that the micromixer with the ratio of the height of the convergence-divergence structure to that of the channel of 0.75 had the best mixing efficiency of over 95% for a wide range of *Re* (*Re* ≥ 5 or *Re* ≤ 0.5). In addition, they used the zigzag channel based on topology optimization to replace the serpentine one to develop a new micromixer [106], and it owned a mixing efficiency of over 93% for a wide range of *Re* (*Re* ≥ 5 or *Re* ≤ 0.5). 

### 3.2. 3D Passive Micromixers

3D passive micromixers are often dependent on complex spatial structures, which require cumbersome fabrication and can generate various vortices such as second flow vortices, Dean vortices, and chaotic advection etc., to enhance mixing.

#### 3.2.1. Lamination Based Micromixers

**Figure 16:**
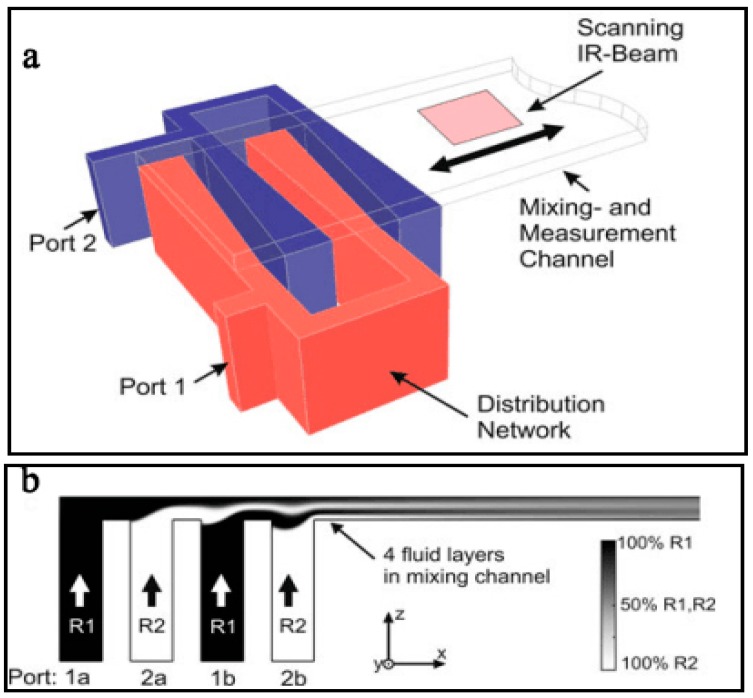
(**a**) Schematic of the lamination based micromixer with four lamination layers; (**b**) Two dimensional flow simulation of the micromixer. Reproduced with permission from [139].

Lamination based micromixers usually comprise multilayer structures, and can achieve excellent mixing in milliseconds. One typical lamination based micromixer was first reported by Branebjerg et al. [138]. As shown in Figure 16, Buchegger et al. [139] presented an aclinic multi-lamination based micromixer with wedge shaped vertical fluid inlets for fast and efficient mixing. In this micromixer, two ports with 10 μm width were split into four vertical inlets through the distribution network. Then, 4 fluid layers were formed in the mixing channel to increase the contact area. The simulation results showed that the mixing efficiency could reach 90% in 0.64 ms under a diffusion coefficient of 2 × 10^−9^ m^2^/s. Proton exchange reaction of H_2_O and D_2_O forming 2 HDO was well achieved. Similarly, SadAbadi et al. [140] designed a simple 3-layer micromixer with a high mixing efficiency of 85% for *Re* < 5.5. 

**Figure 17:**
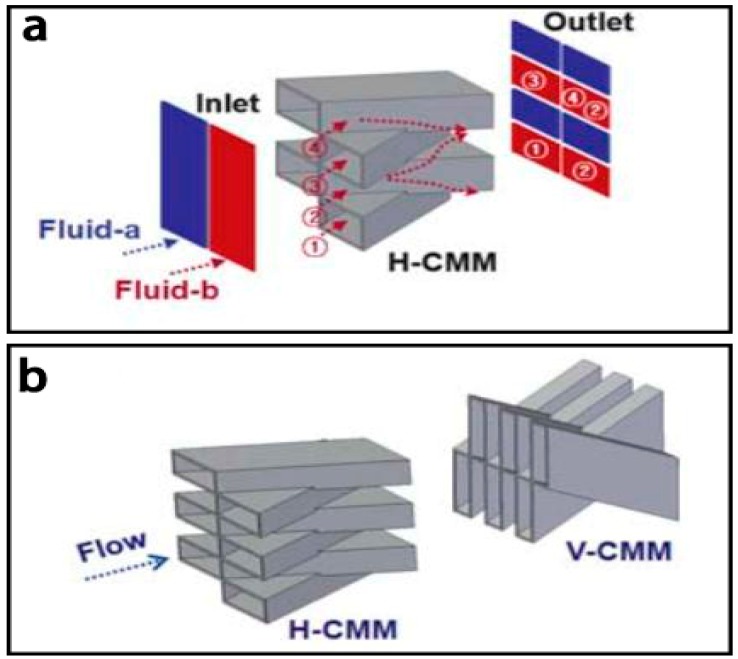
(**a**) Schematic of the lamination based micromixer with crossing manifold structure; (**b**) The horizontally crossing manifold micromixer (H-CMM) and the vertically crossing manifold micromixers (H/V-CMM). Reproduced with permission from [141].

Lim et al. [141] proposed another lamination based micromixer, also called a crossing manifold micromixer (CMM) (Figure 17). It had a three-dimensional microstructure with a sequential configuration of horizontally and vertically crossing tube bundles. In this micromixer, two fluids were rearranged alternately in vertical and horizontal direction for fast mixing by two kinds of mixing modules: horizontally crossing manifold micromixer (H-CMM) and vertically crossing manifold micromixer (V-CMM), respectively. According to the simulation, when V-CMM was set at the distance of 50 mm from H-CMM, the mixing efficiency of 90% could be estimated in the channel length of 250 mm with a total flow rate of 0.003 mL/min.

#### 3.2.2. Chamber Based Micromixers

Chambers with special structures, such as a convergence–divergence structure, recirculation structure and counterflow structure, are often used to significantly improve the mixing efficiency in passive micromixers.

**Figure 18:**
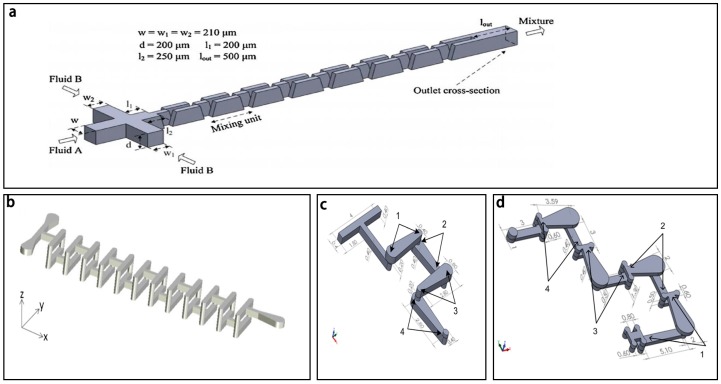
(**a**) Schematic of the chamber based micromixer with shifted trapezoidal chambers; (**b**) Schematic of the H micromixer; (**c**) Schematic of the chain micromixer; (**d**) Schematic of the H-C micromixer. Reproduced with permission from [111,144].

One typical chamber based micromixer, using a chamber with convergence-divergence structure was proposed by Hai et al. [107]. Based on the effect of stretching-folding in both vertical and horizontal directions, this convergence-divergence structure was designed as trapezoidal shape to provide a high mixing efficiency for low flowrate fluids. Simulation results showed that it retained a high mixing efficiency of over 80% for a low Reynolds number of between 0.5 and 60 with a total mixing length of 3870 μm. Combining this micromixer with the unbalanced splits and cross-collisions of fluids, an improved micromixer with shifted trapezoidal chambers and a total mixing length of 5000 μm was then presented and shown in Figure 18a [109]. Simulation results showed that the mixing efficiency was over 80% for an entire range of Reynolds numbers from 0.5 to 100. Recently, a novel SAR micromixer, namely the H-C mixer, combining both the H mixer [142] and the Chain mixer [143] (Figure 18c,d), was developed by Viktorov et al. [111] (Figure 18b). Fluid folding, rotation and expansion occurred in the channel with the splitting-recombination and convergence–divergence structures, thus resulting in a good mixing efficiency of over 93%. The H-C mixer could be considered for industrial applications due to its simple manufacturing procedure, great mixing efficiency and low pressure drop.

**Figure 19:**
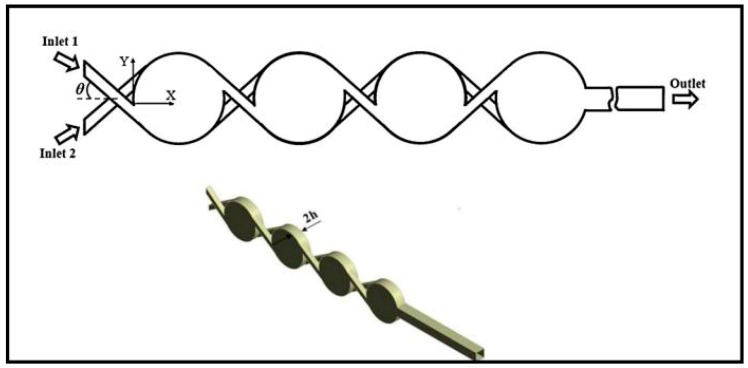
Schematic of the chamber based micromixers with circular chambers. Reproduced with permission from [110].

Another typical chamber based micromixer with circular chambers has already been reported [145,146,147] and circular chambers have been found to be effective in mixing over a wide range of Reynolds numbers. Alam et al. [110] proposed a chamber based micromixer with eight circular chambers and two constriction channels to connect the adjacent chambers (Figure 19). Simulation results showed that this micromixer could achieve a mixing efficiency of 88% at a low Reynolds number (*Re* = 0.1) where diffusion dominated the fluidic mixing.

#### 3.2.3. 3D Spiral Based Micromixers

**Figure 20:**
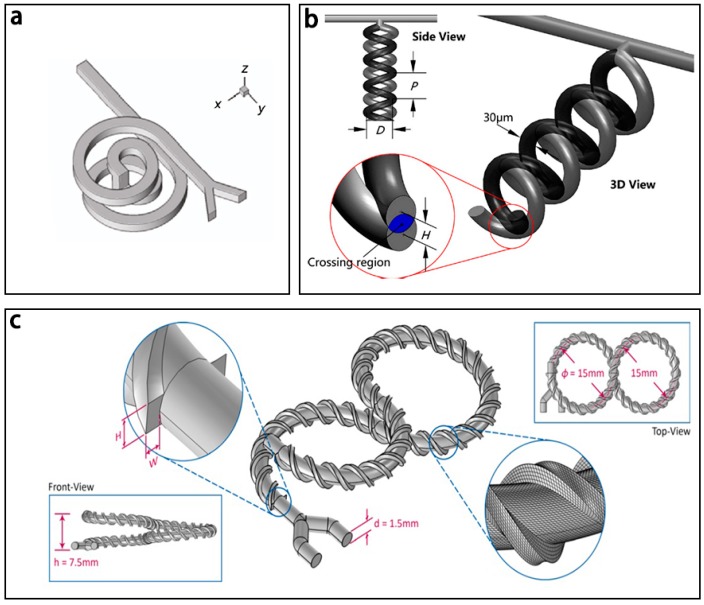
Schematic of the 3D spiral based micromixer (**a**) with two spiral microchannels and an erect channel; (**b**) with double helical channels in opposite directions; and (**c**) with 3D spiral and fine-threaded microchannel. Reproduced with permission from [112,113,148].

One typical 3D spiral based micromixer with two spiral microchannels and an erect channel was presented by Yang [112] (Figure 20a). For *Re* > 40, the maximum mixing efficiency could be up to 90% and the erect channel played a significant role in mixing. 

Recently, Liu et al. [113] proposed a novel 3D spiral based micromixer (Figure 20b), which consists of double helical channels in opposite directions to create repeated crossing regions. The simulation and experimental results showed that the micromixer had a high mixing efficiency of 99% for a wide range of low *Re* (0.003–30). In addition, Rafeie et al. [148] presented an effective micromixer (mixing efficiency > 90%) which incorporates the 3D spiral and fine-threaded microchannels for a wider range of *Re* (1–1000) (Figure 20c).

#### 3.2.4. Overbridge Based Micromixers

Overbridge based micromixers, often with 3D structures connected by a bridge-shaped channel, are mainly based on the concept of splitting and recombination. 

**Figure 21:**
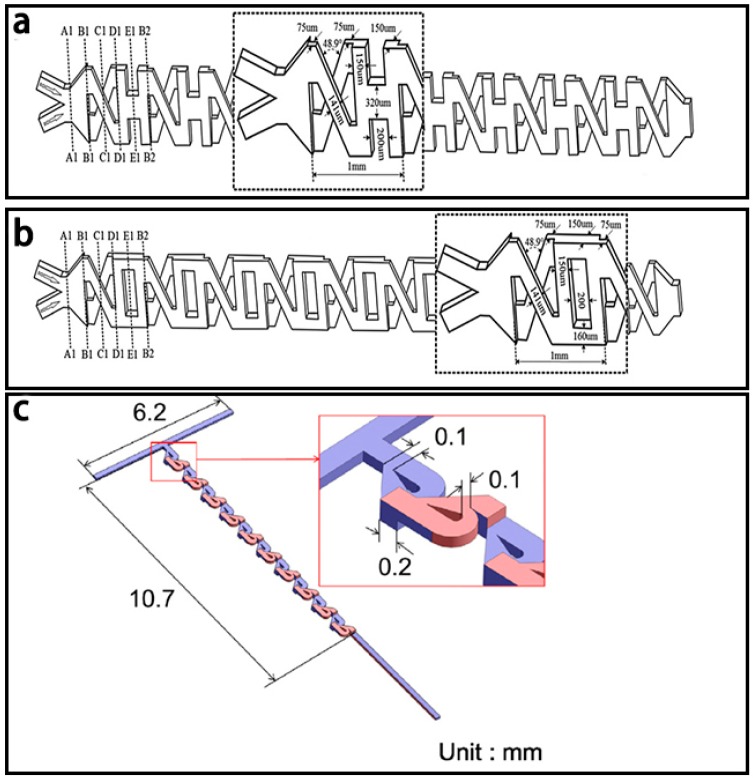
Schematic of the overbridge based micromixer (**a**) with XH-shaped structures; (**b**) with XO-shaped structures; and (**c**) with Tesla structures. Reproduced with permission from [116,117].

One typical overbridge based micromixer with splitting channels of unequal widths was proposed by Li et al. [115] and it had a high mixing efficiency of over 90% for a *Re* range from 0.01 to 200 according to the simulation results and the same mixing efficiency for a *Re* range from 0.01 to 50 by the experimental results. And the mixing efficiencies with different inlet flow rates ranging from a ratio (fluid 1: fluid 2) of 1:9 to 9:1 were also compared and it showed that the best mixing efficiency (100%) was reached with the ratio of 1:9 at *Re* = 0.01. Feng et al. [117] used the X-shaped structures to connect the O-shaped structures or H-shaped structures in passive micromixers with total length of 10.25 mm (Figure 21a,b). Experimental results showed that the mixing efficiency of the micromixer with X-shaped structures and H-shaped structures and the micromixer with X-shaped structures and O-shaped structures were from 91.8% to 87.7% and 89.4% to 72.9% respectively when the *Re* increases from 0.3 to 60. 

Based on the well-known Tesla structures, another overbridge based micromixer was fabricated by Yang et al. [116] (Figure 21c). Binding reactions between the antibodies and the antigens for the detection of cancer cells were efficiently realized by this micromixer. By placing Tesla structures on other Tesla structures, this micromixer could realize a mixing efficiency of 94% for a *Re* range from 0.1 to 100. Simulation results also showed that a larger contact area between two Tesla structures led to better mixing. 

## 4. Conclusion and Future Trends

Micromixing has made rapid developments over the past decade due to advances in MEMS and Microfluidics. Compared with conventional macro-scale mixers, both passive and active micromixers have demonstrated their features of faster mixing, easier fabrication, higher efficiency, and lower cost. This paper systematically reviewed the recent advances in various active and passive micromixers. The micromixers with different structures and external fields were discussed and their advances and defects were also pointed out for reference. 

With the increasing needs from the biomedical, agricultural, food and environmental fields, microfluidic chips for rapid and automatic screening or monitoring of such biological and chemical targets as glucose, pathogens and melamine, etc. have attracted more and more attention, which require the micromixing technologies to boost the on-chip biochemical detection assays. Thus, the integration of micromixers with biochemical sensors will be a promising trend. In recent years, 3D printing technology—with an accuracy of up to several micrometers and with the incorporation of various materials—has been widely used for the development of complex structures and the fabrication of various valves and pumps in a very short time [149]. The use of 3D printing technology to fabricate micromixers with complex structures more accurately, easily and faster at lower cost is very promising. Additionally, paper-channel micromixers combined with external fields have the potential to provide simple, low-cost and disposable methods for point-of-care diagnostics. 

## Figures and Tables

**Table 1:** Active micromixers reported in recent five years.

Energy Source	Characteristic	Mixing Time (s)	*Re*	Mixing Efficiency	Reference
Electrical field	Conductive sidewall	0.033	<1	-	[21] ^b^
	Ferrofluid flow	20	-	-	[23] ^c^
	Circular copper electrodes	110	-	>90%	[20] ^b^
	Asymmetric lateral structure	-	-	100% ^2^	[26] ^a^
	Floating electrode	1.15	-	95% ^3^	[25] ^c^
Pressure field	Pulse width modulation	-	83–125	90% ^2^	[15] ^a^
	Braille Pin Actuator	0.5	4	90% ^2^	[14] ^b^
	Rotary peristaltic micropump	1	-	90% ^4^	[7] ^c^
	Single-chamber micropumps	0.45	0.03–30	92% ^2^	[16] ^a^
Magnetic field	Permanent magnet	80	-	90% ^2^	[57] ^c^
	Magnetohydrodynamic Actuation	-	0.12	81% ^1^	[47] ^a^
	Rotating magnetic field	-	-	90–92% ^2^	[52] ^c^
	Hybrid gradient magnetic field	8	-	97–99% ^4^	[48] ^a^
	rotating magnetic microbeads	2.5–9	-	-	[54] ^c^
Sound field	Bubble cavitation	0.100	0.01	92% ^2^	[38] ^b^
	Vibrating membrane	0.003	-	90% ^3^	[6] ^c^
	Bubbles	0.05	0.01	93% ^2^	[43] ^b^
	Micro-pillars	6	-	-	[40] ^b^
	Sharp-edges	0.18	-	-	[36] ^b^

^a^ Research including only simulated results. ^b^ Research including only experimental results. ^c^ Research including both of simulated and experimental results. ^1^ Mixing Index calculated based on Equation (1). ^2^ Mixing Index calculated based on Equation (2). ^3^ Mixing Index calculated based on Equation (3). ^4^ Mixing Index calculated based on Equation (4).

**Table 2:** Active micromixers reported in recent five years.

Dimension	Structure	Characteristic	*Re*	Mixing Length (μm)	Mixing Efficiency	Reference
2D	Unbalanced collisions channel	Unbalanced three-split recombine sub-channels	30–80	8275	90% ^3^	[95] ^a^
		Dislocation structure	<80	8000	85% ^2^	[96] ^c^
	Embedded Barriers channel	Triangle baffle	1	6400	85.5% ^3^	[97] ^c^
		Curved micromixers with cylindrical obstructions	0.1–60	8280	88% ^3^	[98] ^a^
	Spiral	Single logarithmic spiral	67	12,000	86% ^4^	[99] ^c^
		Double logarithmic spirals	50	5000	80% ^3^	[100] ^a^
	Convergent–divergent channel	Sigma channel	0.91	8000	79.1% ^3^	[101] ^a^
		Semi-elliptical walls	35.5	-	80% ^2^	[102] ^c^
		Convergent–divergent walls	10–70	6720	90% ^3^	[103] ^a^
		Ellipse-like micro-pillars	≤1	9000	80% ^2^	[104] ^c^
		Reversed flow in square wave channel	≤0.1 or ≥10	3710	95% ^2^	[105] ^a^
		Reversed flow in zigzag channel	≤0.5 or ≥5	-	93% ^2^	[106] ^a^
3D	Chamber	Trapezoidal chambers	0.5–60	3870	80% ^2^	[107] ^a^
		Trapezoidal-zigzag channels	0.1–0.9 or 20–80	3610	90% ^3^	[108] ^a^
		Unbalanced split and cross-collision chambers	0.5–100	5000	80% ^2^	[109] ^c^
		Circular mixing chambers	0.1	6400	88%^3^	[110] ^a^
		Split and recombine chambers	1–100	-	90% ^3^	[111] ^c^
	3D Spiral	Three dimensional spirals	40	2340	90% ^1^	[112] ^c^
		Cross-linked dual helicals	0.003–30	320	99% ^2^	[113] ^a^
		Tapered structures	50	10,500	90% ^3^	[114] ^c^
	Overbridge	Overbridge-shaped channel	0.01–50	2000	90% ^4^	[115] ^c^
		Tesla structures	0.1–100	10,700	94% ^2^	[116] ^c^
		X-shape structures combined with H-shape structures	0.3–60	102,500	87.7% ^3^	[117] ^c^
		X-shape structures combined with O-shape structures	0.3–60	102,500	72.9% ^3^	[117] ^c^
		Serpentine crossing channels	0.2–10	7500	99% ^3^	[118] ^c^

^a^ Research including only simulated results. ^b^ Research including only experimental results. ^c^ Research including both of simulated and experimental results. ^1^ Mixing Index calculated based on Equation (1). ^2^ Mixing Index calculated based on Equation (2). ^3^ Mixing Index calculated based on Equation (3). ^4^Mixing Index calculated based on Equation (4).
